# Veränderungen des Rauschtrinkens bei Jugendlichen und jungen Erwachsenen in Deutschland in Abhängigkeit von Bildungsniveau und Migrationshintergrund

**DOI:** 10.1007/s00103-021-03332-x

**Published:** 2021-05-22

**Authors:** Boris Orth, Christina Merkel

**Affiliations:** Referat 2-25 Forschung, Qualitätssicherung, Bundeszentrale für gesundheitliche Aufklärung (BZgA), Maarweg 149–161, 50825 Köln, Deutschland

**Keywords:** Rauschtrinken, Trends, Jugendliche und junge Erwachsene, Bildungsniveau, Migrationshintergrund, Binge drinking, Trend, Young people, Level of education, Migration background

## Abstract

**Hintergrund:**

Studien zeigen, dass die Verbreitung des Rauschtrinkens unter jungen Menschen in Deutschland insgesamt rückläufig ist. Diese Veränderung wird in der Regel in Abhängigkeit von Alter und Geschlecht näher untersucht. Dieser Beitrag vertieft diese Analysen und untersucht, ob sich der Rückgang des Rauschtrinkens junger Menschen in Abhängigkeit von Bildungsniveau und Migrationshintergrund unterscheidet.

**Methoden:**

Auf Grundlage von Repräsentativbefragungen der Bundeszentrale für gesundheitliche Aufklärung (BZgA) wurden für den Zeitraum 2008 bis 2019 für männliche und weibliche 12- bis 17-jährige Jugendliche und 18- bis 25-jährige junge Erwachsene die 30-Tage-Prävalenzen des Rauschtrinkens ermittelt. Mit logistischen Regressionsanalysen wurden Trendverläufe für den Zeitraum 2008 bis 2019 geschätzt. Dies erfolgte auch in Abhängigkeit von Bildungsniveau und Migrationshintergrund.

**Ergebnisse:**

In einen Alkoholrausch trinken sich, über alle Befragungen gesehen, mehr junge Erwachsene als Jugendliche, mehr männliche als weibliche junge Menschen und mehr junge Menschen ohne einen Migrationshintergrund. Im Zeitraum 2008 bis 2019 ging die 30-Tage-Prävalenz des Rauschtrinkens bei Jugendlichen (männlich: von 23,0 % auf 16,4 %; weiblich: von 17,7 % auf 10,7 %) sowie jungen Männern (von 53,0 % auf 43,9 %) insgesamt gesehen zurück, bei jungen Frauen veränderte sie sich statistisch nicht signifikant (2008: 28,1 %; 2019: 24,5 %). Die Trendanalysen in Abhängigkeit von Bildungsniveau und Migrationshintergrund zeigen, dass zumindest bei jungen Frauen ohne (Fach‑)Abitur ein Rückgang des Rauschtrinkens erfolgt.

**Diskussion:**

Der Rückgang des Rauschtrinkens kann sich in Abhängigkeit von sozialen Merkmalen unterscheiden. Solche Unterschiede sollten in der Prävention des Rauschtrinkens berücksichtigt werden. Insbesondere junge Frauen mit höherem Bildungsniveau müssen mit Präventionsangeboten erreicht werden.

## Abstract (en)

**Background:**

Studies show that the prevalence of binge drinking among young people in Germany is declining overall. This change is usually studied in more detail based on age and gender. This paper expands on these analyses and examines whether the decline in binge drinking among young people differs as a function of educational level and migration background.

**Methods:**

Based on representative surveys conducted by the Federal Center for Health Education (BZgA), 30-day prevalences of binge drinking were determined for Germany between 2008 and 2019 for male and female 12- to 17-year-old adolescents and 18- to 25-year-old young adults. Logistic regression analyses were used to estimate trend patterns for the period from 2008 to 2019. This was also done as a function of education level and migration background.

**Results:**

Across all surveys, more young adults than adolescents, more male than female young people, and more young people without a migration background get drunk. Between 2008 and 2019, the 30-day prevalence of binge drinking decreased overall among adolescents (male: from 23.0 to 16.4%; female: from 17.7 to 10.7%) and young men (from 53.0 to 43.9%), and it did not change statistically significantly among young women (2008: 28.1%; 2019: 24.5%). The trend analyses depending on the level of education and migration background show that at least among young women without a (technical) higher education entrance qualification there is a decrease in binge drinking.

**Discussion:**

The decline in binge drinking may differ depending on social characteristics. Such differences should be taken into account in the prevention of binge drinking. In particular, young women with a higher level of education must be reached with prevention offers.

## Einleitung

Alkoholkonsum schädigt das Gehirn und das Nervensystem, verursacht Erkrankungen der Leber, des Herz-Kreislauf-Systems sowie anderer Organe und kann zu einer Abhängigkeitserkrankung führen [1]. Aufgrund seiner weiten Verbreitung in vielen Regionen dieser Welt [2] ist der Konsum von Alkohol einer der global führenden Risikofaktoren für schwerwiegende Krankheiten sowie vorzeitigen Tod [3].

Junge Menschen sind aufgrund ihres noch nicht ausgereiften Organismus besonders vulnerabel für negative gesundheitliche Konsequenzen durch Alkoholkonsum. Alkohol kann die gesunde Entwicklung des Gehirns, das im Jugendalter noch ausreift, beeinträchtigen [4–6] und erhöht zudem das Risiko für alkoholbezogene und andere psychische Störungen sowie für soziale und Entwicklungsprobleme im Jugendalter [7]. Um dem vorzubeugen, wird Jugendlichen empfohlen, Alkohol weitgehend zu meiden [8] oder besser gar keinen Alkohol zu trinken [9].

Auch Erwachsenen wird empfohlen, Alkohol maßvoll zu konsumieren. Männer sollten nicht mehr als 24 g Alkohol am Tag (das entspricht etwa 0,5–0,6 l Bier) und Frauen nicht mehr als 12 g Alkohol pro Tag konsumieren [8]. Aber auch ein moderater Alkoholkonsum ist mit einem erhöhten Risiko für Erkrankungen verknüpft [9]. Männer und Frauen sollten außerdem auf Rauschtrinken verzichten [8, 9]. Beim Rauschtrinken, d. h. dem Konsum größerer Mengen Alkohol innerhalb eines kürzeren Zeitraums, wird die Blutalkoholkonzentration soweit erhöht (z. B. 0,8 Promille [10]), dass es zu einem Rauschzustand kommen kann. International wird Rauschtrinken häufig definiert als der Konsum von 5 (bei Frauen manchmal 4 [10, 11]) oder mehr alkoholischen Getränken bei einer Trinkgelegenheit [12].

Folgen des Rauschtrinkens können unter anderem Angstzustände, Verletzungen, Verkehrsunfälle, aktive wie passive Gewalt sowie Alkoholvergiftungen sein [13]. Im Jahr 2018 wurden in Deutschland knapp 20.500 Kinder, Jugendliche und junge Erwachsene zwischen 10 und 20 Jahren wegen eines akuten Alkoholrauschs stationär im Krankenhaus behandelt [14]. Rauschtrinken ist zudem mit einem erhöhten Risiko für Katersymptome, Gedächtnisverlust, Versäumen von Lehrveranstaltungen, Lernrückständen und Streitereien mit Freunden verbunden [15].

Manche Kinder und Jugendliche sind besonders anfällig für riskante Alkoholtrinkmuster wie dem Rauschtrinken. Studien deuten darauf hin, dass beispielsweise Kinder mit frühen psychischen Störungen [16] und Kinder aus suchtbelasteten Familien [17] bzw. aus Familien, in denen das Familienklima gestört ist und/oder psychische Erkrankungen bei den Eltern vorliegen [18], ein höheres Risiko für riskanten Alkoholkonsum im Jugend- und jungen Erwachsenenalter aufweisen. Gleichwohl muss beachtet werden, dass die skizzierten Gruppen heterogen sind, einzelne Jugendliche unterschiedlich resilient sein können und es weitere Faktoren gibt, die günstig oder ungünstig auf das Trinkverhalten einwirken können.

In der Forschung zum Gesundheitsverhalten junger Menschen werden 3 allgemeine Bereiche unterschieden, die den Substanzkonsum junger Menschen beeinflussen. Einen Einfluss haben erstens gesellschaftlich-kulturelle Faktoren wie gesetzliche Regelungen zur Verfügbarkeit von Alkohol oder Werbung, zweitens interpersonell-soziale Faktoren wie das Konsumverhalten von Eltern oder Freunden und drittens intrapersonale Faktoren wie das Temperament oder substanzbezogene Einstellungen [19, 20].

Repräsentativbefragungen, die in Deutschland bundesweit oder regional zum Alkoholkonsum durchgeführt wurden, zeigen, dass Rauschtrinken mit sozialen Merkmalen zusammenhängt. So ist Rauschtrinken unter jungen Menschen weiter verbreitet, wenn sie männlich [21–24] oder älter sind [21, 22, 25, 26], keinen Migrationshintergrund haben [21, 25] oder nicht das Gymnasium besuchen [23].

Diese Repräsentativbefragungen werden wiederholt durchgeführt und differenzieren in den Trendanalysen in der Regel nach Alters- und Geschlechtergruppen. Auch der letzte Forschungsbericht der Bundeszentrale für gesundheitliche Aufklärung (BZgA; [21]) unterscheidet in den Trends ausschließlich nach diesen Merkmalen. Andere Merkmale wie das Bildungsniveau und der Migrationshintergrund, die in Zusammenhang mit dem Rauschtrinken stehen können, werden nicht berücksichtigt. Internationale Studien zeigen aber, dass es sich lohnen kann, die Trends nach weiteren sozialen Merkmalen aufzuschlüsseln [27]. Wenn sich auch für Deutschland Teilgruppen nach weiteren sozialen Merkmalen finden lassen, in denen sich das Rauschtrinken unterschiedlich verändert, könnte ein solcher Befund Hinweise dafür liefern, wie Maßnahmen zur Förderung eines verantwortungsvollen Umgangs mit Alkohol noch zielgruppengerechter auszugestalten sind.

Ziel dieses Beitrags ist es deshalb, die bisherigen Trendanalysen der BZgA zu vertiefen und innerhalb der einzelnen Alters- und Geschlechtergruppen zusätzlich nach Bildungsniveau und Migrationshintergrund zu unterscheiden. Damit wird untersucht, wie sich das Rauschtrinken bei stärker differenzierten sozialen Teilgruppen junger Menschen im Zeitraum von 2008 bis 2019 in Deutschland entwickelt.

## Methoden

### Studien, Studiendesigns und Stichproben

Dieser Beitrag beruht auf den bundesweiten Repräsentativbefragungen zum Substanzkonsum Jugendlicher und junger Erwachsener in Deutschland, die die BZgA im Zeitraum von 2008 bis 2019 durchgeführt hat: den Drogenaffinitätsstudien der Jahre 2008, 2011, 2015 und 2019 sowie den Alkoholsurveys der Jahre 2010, 2012, 2014, 2016 und 2018. Mit den Drogenaffinitätsstudien untersucht die BZgA seit 1973 den Konsum von Alkohol, illegalen Drogen und das Rauchverhalten Jugendlicher und junger Erwachsener. Die Alkoholsurveys begleiten seit 2010 die BZgA-Kampagne „Alkohol? Kenn dein Limit.“ für Jugendliche und junge Erwachsene und vertiefen das Thema Alkohol. In allen 9 Erhebungen sind die Kernfragen zum Alkoholkonsum im Wesentlichen gleich. Dieser Beitrag konzentriert sich auf die Daten ab dem Jahr 2008, weil seitdem erstmalig für alle Altersgruppen der Migrationshintergrund erfasst wurde.^1^

Das Alter der Befragten reicht in allen 9 Erhebungen von 12 bis 25 Jahre. Die Datenerhebungen erfolgten durch computergestützte Telefoninterviews. Die Grundgesamtheit bildeten dabei von 2008 bis 2012 alle Personen im Alter von 12 bis 25 Jahren, deren Haushalt über einen Festnetzanschluss telefonisch erreichbar war (Festnetzstichprobe) und deren Kenntnisse der deutschen Sprache für die Durchführung des Interviews ausreichten. Die Stichprobenziehungen erfolgten nach dem Gabler-Häder-Design [28]. Seit dem Jahr 2014 wurden die Studien im Dual-Frame-Ansatz durchgeführt, d. h., 30 % der Gesamtstichprobe wurde aus dem Auswahlrahmen der Mobiltelefonnummern gezogen und über das Mobiltelefon befragt [29]. Um methodisch konstant zu bleiben und zur besseren Vergleichbarkeit mit den Festnetzstichproben der Jahre 2008 bis 2012, wurden für diesen Beitrag auch aus den Jahren 2014 bis 2019 ausschließlich die Daten der Festnetzstichproben verwendet [21]. Die Ausschöpfungsquoten der einzelnen Festnetzstichproben lagen zwischen 68,4 % (Drogenaffinitätsstudie 2008) und 40,3 % (Alkoholsurvey 2014). Die Datenerhebungen wurden in den Jahren 2008, 2011, 2015, 2016, 2018 und 2019 von forsa, Gesellschaft für Sozialforschung und statistische Analysen mbH, Berlin und in den Jahren 2010, 2012 und 2014 von der KantarHealth GmbH, München durchgeführt.

### Rauschtrinken und weitere Indikatoren

#### 30-Tage-Prävalenz des Rauschtrinkens.

Rauschtrinken wird in den Befragungen der BZgA seit 2004 als Konsum von 5 oder mehr alkoholischen Getränken bei einer Trinkgelegenheit definiert. Befragte, die angaben, in den letzten 30 Tagen Alkohol getrunken zu haben, wurden gefragt: „An wie vielen dieser Tage haben Sie 5 und mehr Gläser Alkohol hintereinander getrunken?“ Die 30-Tage-Prävalenz des Rauschtrinkens ist in diesem Beitrag definiert als der prozentuale Anteil derer, die an mindestens einem der letzten 30 Tage 5 und mehr Gläser Alkohol hintereinander getrunken haben.^2^

#### Bildung.

Der Indikator zur Bildung ist zweistufig. Die Gruppe der 12- bis 17-jährigen Jugendlichen wurde unterteilt in Jugendliche, die Haupt‑, Gesamt‑, Real- oder sonstige Schulen besuchen, in Ausbildung sind oder keine Angaben zur Schulform machen, und in Jugendliche, die das Gymnasium besuchen.

Die Gruppe der 18- bis 25-jährigen jungen Erwachsenen wurde unterteilt in junge Erwachsene, die maximal einen Realschulabschluss haben oder nicht im Gymnasium sind, falls sie noch zur Schule gehen, und in junge Erwachsene, die mindestens Fachabitur haben oder das Gymnasium besuchen, falls sie noch Schülerin oder Schüler sind.

#### Migrationshintergrund.

Der Migrationshintergrund wurde über Angaben zum Geburtsland und zur Staatsangehörigkeit der Befragten selbst und ihrer Eltern bestimmt [30]. Ein Migrationshintergrund ist gegeben, wenn der bzw. die Befragte selbst oder mindestens ein Elternteil in einem anderen Land als Deutschland geboren ist oder eine andere als die deutsche Staatsangehörigkeit hat. Fälle mit unvollständigen Angaben wurden der Gruppe mit Migrationshintergrund zugeordnet.

### Statistische Analysen

Die Datensätze aller Erhebungen wurden für die statistischen Analysen gepoolt und es wurde jeweils eine dichotome Variable für die Bildung (0 = nicht im Gymnasium bzw. kein (Fach‑)Abitur, 1 = im Gymnasium bzw. (Fach‑)Abitur) und den Migrationshintergrund (0 = kein Migrationshintergrund; 1 = mit Migrationshintergrund) gebildet. Für die Gruppen der 12- bis 17-jährigen männlichen und weiblichen Jugendlichen sowie der 18- bis 25-jährigen jungen Männer und Frauen wurden für alle Erhebungsjahre Punktschätzungen mit 95 %-Konfidenzintervallen (95 %-KI) für die 30-Tage-Prävalenzen des Rauschtrinkens berechnet. Diese Kennwerte wurden jeweils auch für die 4 Untergruppen, die sich durch die Kombination der dichotomen Variablen zur Bildung und zum Migrationshintergrund ergeben, bestimmt.

Zur Schätzung von zeitlichen Trends und Veränderungen wurden logistische Regressionsanalysen [31] mit der binären, abhängigen Variable für Rauschtrinken und der stetigen, unabhängigen Variable Erhebungsjahr berechnet (Modell 1). Um unterschiedliche Verläufe in Abhängigkeit von Bildung und Migrationshintergrund zu untersuchen, wurden diese beiden Einflussgrößen zusätzlich in das Regressionsmodell aufgenommen und als Modell mit allen Interaktionseffekten modelliert (Modell 2). Mit dem Bayes’schen Informationskriterium (BIC) wurde beurteilt, mit welchem Modell die beste Anpassung an die beobachteten Werte gelingt. Um die Interpretation der Ergebnisse der logistischen Regressionsanalysen für Modell 2 zu erleichtern, wurden deren Logits in prozentuale Prävalenzwerte transformiert und als Trendlinien über die Zeit grafisch dargestellt [32].

Die Daten wurden durch einen Gewichtungsfaktor an die Alters‑, Geschlechter- und regionale Verteilung der amtlichen Statistiken der einzelnen Jahre angepasst. Das Datenmanagement und die Datenanalysen wurden mit IBM SPSS Statistics Version 26.0.0.0 durchgeführt.

## Ergebnisse

### Stichprobenmerkmale von 2008 bis 2019

An den 9 Erhebungen im Zeitraum 2008 bis 2019 haben insgesamt 44.524 Jugendliche und junge Erwachsene im Alter von 12 bis 25 Jahren teilgenommen. Die weiblichen Befragten, für die in der Befragung 2012 nur Daten zum Rauschtrinken definiert als Konsum von 4 Gläsern Alkohol und mehr vorliegen, wurden von den Analysen zum Rauschtrinken komplett ausgeschlossen (*n* = 2491). Von den verbliebenen 42.033 Befragten wurden weitere 395 Befragte (0,9 %) wegen fehlender Angaben zum Rauschtrinken (weiß nicht, keine Angabe) ausgeschlossen. Damit beruhen die Auswertungen zum Rauschtrinken im Zeitverlauf auf den Angaben von insgesamt 41.638 jungen Menschen.

In Tab. 1 sind für jedes Erhebungsjahr die jeweiligen Fallzahlen männlicher und weiblicher 12- bis 17-jähriger Jugendlicher und 18- bis 25-jähriger junger Männer und Frauen dargestellt. Die Fallzahlen variieren in den einzelnen Zellen von *n* = 602 (männliche Jugendliche im Jahr 2008) bis *n* = 2208 (junge Frauen im Jahr 2010). Der Anstieg der Fallzahlen zwischen 2008 und 2010 ist darauf zurückzuführen, dass ab 2010 die Gesamtzahl der Festnetzstichproben von rund 3000 auf rund 5000 Befragte erhöht wurde.

**Table 1:** 

			2008	2010	2011	2012	2014	2015	2016	2018	2019
Männliche Jugendliche	Insgesamt	*n*	602	1288	1032	989	1043	1273	1265	1304	1396
	Kein Gymnasiast/kein Migrationshintergrund	%	41,7	41,3	40,3	40,6	39,2	38,8	36,0	35,7	35,9
	Gymnasiast/kein Migrationshintergrund	%	34,5	31,0	40,3	32,6	31,1	40,7	37,7	44,6	46,0
	Kein Gymnasiast/mit Migrationshintergrund	%	17,1	18,0	11,7	16,9	19,1	13,0	14,4	10,4	8,2
	Gymnasiast/mit Migrationshintergrund	%	6,8	9,6	7,7	9,9	10,6	7,4	11,9	9,4	9,9
Weibliche Jugendliche	Insgesamt	*n*	595	1268	992	1029	962	1168	1162	1243	1305
	Kein Gymnasiast/kein Migrationshintergrund	%	38,4	33,4	37,4	34,6	31,9	32,6	32,2	31,8	27,0
	Gymnasiast/kein Migrationshintergrund	%	35,1	36,6	44,3	38,7	39,0	43,1	41,9	48,0	55,3
	Kein Gymnasiast/mit Migrationshintergrund	%	17,5	19,1	10,0	16,6	20,4	13,0	15,3	8,8	7,4
	Gymnasiast/mit Migrationshintergrund	%	9,0	10,9	8,4	10,2	8,8	11,2	10,6	11,4	10,3
Junge Männer	Insgesamt	*n*	867	2187	1477	1497	1496	1292	1373	1303	1190
	Ohne (Fach‑)Abitur/kein Migrationshintergrund	%	33,8	32,3	28,0	29,9	23,3	25,1	25,1	20,1	26,3
	(Fach‑)Abitur/kein Migrationshintergrund	%	43,7	43,3	53,9	48,7	53,2	52,3	51,4	62,5	57,9
	Ohne (Fach‑)Abitur/mit Migrationshintergrund	%	12,2	11,5	7,8	8,6	11,2	10,4	9,5	6,4	5,8
	(Fach‑)Abitur/mit Migrationshintergrund	%	10,2	12,9	10,3	12,8	12,3	12,2	14,0	11,0	10,0
Junge Frauen	Insgesamt	*n*	889	2208	1472	1462	1357	1088	1068	1046	941
	Ohne (Fach‑)Abitur/kein Migrationshintergrund	%	32,6	26,5	25,3	23,5	20,8	19,8	15,0	14,1	16,8
	(Fach‑)Abitur/kein Migrationshintergrund	%	45,6	48,0	54,2	51,1	51,6	56,8	56,5	61,5	62,5
	Ohne (Fach‑)Abitur/mit Migrationshintergrund	%	12,6	11,5	8,3	11,3	9,8	7,1	10,6	7,1	4,7
	(Fach‑)Abitur/mit Migrationshintergrund	%	9,2	14,0	12,2	14,1	17,8	16,2	17,9	17,3	16,0

*n* ungewichtete Fallzahlen; % gewichtete Spaltenprozent. Weibliche Jugendliche und junge Frauen der Befragung 2012 werden hier auch aufgeführt, wurden aber bei den Analysen zum Rauschtrinken nicht berücksichtigt, weil ihnen in diesem Jahr nur die Frage nach 4 Gläsern Alkohol oder mehr verwertbar ist

Innerhalb der 4 Alters- und Geschlechtergruppen sind in allen Erhebungen junge Menschen ohne Migrationshintergrund in der Mehrheit. Die Anteile der Gymnasiastinnen und Gymnasiasten bzw. jungen Erwachsenen mit (Fach‑)Abitur, nehmen im Laufe der Jahre zu. Durch die Differenzierung nach Erhebungsjahr, Alter, Geschlecht, Bildung und Migrationshintergrund ergeben sich in manchen Zellen vergleichsweise geringe Fallzahlen. So haben von den jungen Männern, die im Jahr 2019 befragt wurden, 56 kein (Fach‑)Abitur und einen Migrationshintergrund. 708 haben (Fach‑)Abitur und keinen Migrationshintergrund (ungewichtete Fallzahlen, in der Tabelle nicht dargestellt). Dadurch sind die Vertrauensbereiche der Prävalenzschätzungen für die Untergruppen unterschiedlich groß (s. unten).

### 30-Tage-Prävalenzen des Rauschtrinkens von 2008 bis 2019

#### 12- bis 17-jährige Jugendliche

Im Zeitraum von 2008 bis 2019 sind die 30-Tage-Prävalenzen des Rauschtrinkens 12- bis 17-jähriger männlicher und weiblicher Jugendlicher insgesamt zurückgegangen (Tab. 2). In der Gesamtgruppe aller männlichen Jugendlichen verringert sich die Prävalenz von 23,0 % (2008) auf 16,4 % (2019). In der Gruppe der weiblichen Jugendlichen geht sie von 17,7 % (2008) auf 10,7 % (2019) zurück, wobei weibliche Jugendliche schon im Jahr 2011 eine Prävalenz von 10,5 % erreichen. Die nicht überschneidenden Konfidenzintervalle der Jahre 2008 und 2019 deuten an, dass das Rauschtrinken männlicher und weiblicher Jugendlicher statistisch signifikant zurückgegangen ist. In allen Erhebungen ist das Rauschtrinken unter männlichen Jugendlichen weiterverbreitet als unter weiblichen Jugendlichen. Im gesamten Beobachtungszeitraum (letzte Spalte rechts) hat sich etwa jeder 6. männliche Jugendliche (17,9 %), aber nur etwa jede 9. weibliche Jugendliche (11,3 %) in den letzten 30 Tagen vor der Befragung in einen Alkoholrausch getrunken.

**Table 2:** 

			2008	2010	2011	2012	2014	2015	2016	2018	2019	Gesamt
Männlich	Insgesamt	%	23,0	20,4	19,6	18,7	14,6	15,9	16,5	16,9	16,4	17,9
		95 %-KI	19,9–26,5	17,9–23,0	17,4–22,1	16,1–21,6	12,5–16,9	13,6–18,4	14,2–19,0	14,7–19,4	14,2–18,9	17,1–18,8
	*Kein Gymnasiast*	%	31,1	24,4	20,1	20,3	14,7	17,7	17,4	15,1	18,8	19,9
	Kein Migrationshintergrund	95 %-KI	25,7–37,1	20,3–29,0	16,6–24,2	16,2–25,0	11,6–18,5	14,0–22,2	13,7–21,8	11,7–19,3	15,0–23,3	18,5–21,3
	*Gymnasiast*	%	18,5	21,8	20,9	20,0	21,2	15,6	19,2	18,6	16,1	19,0
	Kein Migrationshintergrund	95 %-KI	13,7–24,4	17,5–27,0	17,4–25,0	15,5–25,4	16,7–26,5	12,3–19,7	15,4–23,6	15,2–22,4	12,9–19,8	17,7–20,5
	*Kein Gymnasiast*	%	15,5	10,7	18,7	14,7	7,5	11,0	6,3	19,4	13,1	12,3
	Mit Migrationshintergrund	95 %-KI	9,8–23,6	7,3–15,5	12,8–26,4	9,6–21,8	4,5–12,4	6,5–17,9	3,5–11,0	12,6–28,5	7,5–22,0	10,5–14,2
	*Gymnasiast*	%	15,6	16,3	11,8	14,7	7,1	16,3	17,4	13,4	12,6	13,9
	Mit Migrationshintergrund	95 %-KI	7,6–29,5	10,6–24,3	6,4–20,6	8,6–24,1	3,7–13,2	10,1–25,2	11,3–25,9	7,4–23,1	7,7–19,7	11,7–16,5
Weiblich	Insgesamt	%	17,7	12,8	10,5	–	11,2	8,9	10,3	10,0	10,7	11,3
		95 %-KI	14,7–21,0	10,8–15,0	8,8–12,6	–	9,3–13,5	7,2–11,1	8,4–12,6	8,3–12,1	8,9–12,8	10,6–12,1
	*Keine Gymnasiastin*	%	17,6	13,3	10,5	–	11,2	10,6	12,2	9,6	12,2	12,0
	Kein Migrationshintergrund	95 %-KI	13,1–23,2	10,2–17,3	7,8–14,0	–	8,0–15,6	7,4–14,9	8,7–17,0	6,6–13,7	8,7–17,0	10,7–13,4
	*Gymnasiastin*	%	20,7	15,5	11,6	–	12,7	8,9	12,0	11,7	11,2	12,5
	Kein Migrationshintergrund	95 %-KI	15,5–27,0	12,0–19,7	8,9–14,9	–	9,6–16,6	6,4–12,2	9,0–15,8	9,1–14,9	8,8–14,2	11,4–13,8
	*Keine Gymnasiastin*	%	12,8	8,5	7,1	–	8,2	6,8	5,2	8,9	8,5	8,1
	Mit Migrationshintergrund	95 %-KI	7,7–20,5	4,9–14,5	3,4–14,3	–	4,7–13,8	3,2–14,2	2,7–9,9	4,5–17,0	4,1–16,7	6,5–10,2
	*Gymnasiastin*	%	15,9	9,4	9,2	–	11,8	6,7	5,1	5,3	5,1	8,0
	Mit Migrationshintergrund	95 %-KI	8,5–27,9	5,6–15,5	4,6–17,4	–	6,6–20,4	2,9–14,7	2,4–10,4	2,1–12,8	2,3–10,7	6,3–10,4

Angaben in Prozent (%) mit 95 %-Konfidenzintervallen (95 %-KI). Definition Rauschtrinken: 5 Gläser Alkohol oder mehr bei einer Gelegenheit. Für weibliche Befragte ist in 2012 nur die Frage nach 4 Gläsern Alkohol oder mehr verwertbar. Deshalb sind in 2012 für diese Gruppe keine Ergebnisse dargestellt

Über alle Messzeitpunkte hinweg gesehen zeigen sich insgesamt keine Bildungsunterschiede im Rauschtrinken Jugendlicher. Ein deutlicher Zusammenhang besteht aber mit dem Migrationshintergrund. Rauschtrinken praktizieren mehr Jugendliche ohne Migrationshintergrund als Jugendliche mit Migrationshintergrund. Zumindest in der Gesamtspalte überschneiden sich die Konfidenzintervalle der jeweiligen Vergleichsgruppen nicht (Beispiel: Gymnasiasten ohne Migrationshintergrund 19,0 % [95 %-KI: 17,7–20,5 %]; Gymnasiasten mit Migrationshintergrund 13,9 % [95 %-KI: 11,7–16,5 %]).

#### 18- bis 25-jährige junge Erwachsene

In den Gesamtgruppen der jungen Männer und Frauen (Tab. 3) verläuft die zeitliche Entwicklung des Rauschtrinkens unterschiedlich. Während bei 18- bis 25-jährigen jungen Männern das Rauschtrinken im Zeitraum von 2008 bis 2019 zurückgeht, bleibt es bei 18- bis 25-jährigen jungen Frauen unverändert. Unter jungen Männern ist das Rauschtrinken weiter verbreitet als unter jungen Frauen. Obwohl sich junge Männer durch den Rückgang im Prozentwert den jungen Frauen annähern, ist der Geschlechterunterschied auch 2019 noch erheblich.

**Table 3:** 

			2008	2010	2011	2012	2014	2015	2016	2018	2019	Gesamt
Männlich	Insgesamt	%	53,0	49,5	54,5	52,9	44,0	44,6	42,5	46,3	43,9	47,8
		95 %-KI	49,6–56,3	46,9–52,0	52,0–57,1	49,7–56,0	40,9–47,2	41,4–47,9	39,3–45,8	42,7–49,9	40,2–47,7	46,7–48,9
	*Ohne (Fach‑)Abitur*	%	53,8	49,4	55,2	58,2	42,7	44,7	45,9	51,4	42,7	49,5
	Kein Migrationshintergrund	95 %-KI	47,9–59,5	44,7–54,0	50,3–59,9	52,2–64,0	36,6–48,9	38,7–51,0	38,9–53,0	44,8–58,0	35,1–50,0	47,4–51,6
	*(Fach‑)Abitur*	%	55,5	50,9	55,8	55,2	50,6	46,6	44,8	48,5	48,2	50,3
	Kein Migrationshintergrund	95 %-KI	50,4–60,4	47,0–54,7	52,3–59,2	50,8–59,4	46,1–55,1	42,1–51,2	40,4–49,2	43,7–53,4	43,4–53,0	48,8–51,8
	*Ohne (Fach‑)Abitur*	%	46,7	47,5	48,4	37,6	28,0	36,4	26,2	28,5	22,1	36,8
	Mit Migrationshintergrund	95 %-KI	37,6–56,1	40,2–54,9	39,3–57,5	27,9–48,4	19,6–38,2	27,1–46,7	17,7–37,0	18,5–41,1	12,0–37,0	33,6–40,2
	*(Fach‑)Abitur*	%	47,4	46,7	50,9	41,8	32,7	43,1	39,5	34,6	35,6	41,3
	Mit Migrationshintergrund	95 %-KI	37,4–57,7	39,7–53,9	43,0–58,9	33,5–50,7	25,2–41,3	34-1–52,5	31,8–47,6	26,1–44,1	26,9–45,4	38,5–44,3
Weiblich	Insgesamt	%	28,1	25,9	28,7	–	26,3	24,8	22,6	28,4	24,5	26,1
		95 %-KI	25,3–31,2	23,7–28,2	26,4–31,0	–	23,4–29,4	21,9–27,9	19,6–25,8	24,8–32,3	21,0–28,4	25,0–27,1
	*Ohne (Fach‑)Abitur*	%	28,4	27,6	27,6	–	22,1	19,8	19,5	23,3	18,8	24,2
	Kein Migrationshintergrund	95 %-KI	23,6–33,9	23,3–32,4	23,3–32,4	–	16,7–28,5	14,7–26,0	13,8–26,9	16,6–31,6	13,2–26,2	22,3–26,3
	*(Fach‑)Abitur*	%	31,9	30,7	30,5	–	31,8	29,2	29,8	32,6	29,0	30,6
	Kein Migrationshintergrund	95 %-KI	27,5–36,7	27,5–34,2	27,3–33,8	–	27,5–36,4	25,1–33,7	25,4–34,6	27,7–38,0	24,2–34,3	29,0–32,2
	*Ohne (Fach‑)Abitur*	%	19,0	14,4	29,2	–	13,5	17,2	9,0	7,9	16,2	15,5
	Mit Migrationshintergrund	95 %-KI	12,8–27,2	9,5–21,3	21,7–38,0	–	7,8–22,5	9,4–29,4	4,7–16,6	3,8–15,8	6,2–31,1	13,0–18,4
	*(Fach‑)Abitur*	%	20,9	15,5	22,5	–	22,4	18,8	10,4	26,0	15,1	18,6
	Mit Migrationshintergrund	95 %-KI	13,4–31,1	11,4–20,8	17,0–29,3	–	16,4–29,8	13,5–25,6	6,7–15,7	18,4–35,5	8,8–24,6	16,3–21,1

Angaben in Prozent (%) mit 95 %-Konfidenzintervallen (95 %-KI). Definition Rauschtrinken: 5 Gläser Alkohol oder mehr bei einer Gelegenheit. Für weibliche Befragte ist in 2012 nur die Frage nach 4 Gläsern Alkohol oder mehr verwertbar. Deshalb sind in 2012 für diese Gruppe keine Ergebnisse dargestellt

Insgesamt bestehen über alle Erhebungen gesehen nur geringe Bildungsunterschiede im Rauschtrinken junger Erwachsener (letzte Spalte rechts). Wie bei den Jugendlichen gibt es aber auch bei jungen Erwachsenen ohne und mit Migrationshintergrund Unterschiede im Rauschtrinken. Bei jungen Erwachsenen ohne Migrationshintergrund ist Rauschtrinken weiter verbreitet als bei jungen Erwachsenen mit Migrationshintergrund. Wegen teilweise geringer Fallzahlen unterstützen die Konfidenzintervalle nicht in jedem einzelnen Jahr diesen Befund. Aber insgesamt zeigt sich über alle Messzeitpunkte hinweg deutlich, dass ein Migrationshintergrund mit einer niedrigeren 30-Tage-Prävalenz des Rauschtrinkens einhergeht.

### Trendschätzungen für das Rauschtrinken im Zeitraum 2008 bis 2019

Für die Gesamtgruppen der männlichen und weiblichen Jugendlichen und jungen Erwachsenen stützen die Trendschätzungen die oben genannten Befunde (Tab. 4). Werden innerhalb dieser Gruppen die Trends durch ein einfaches logistisches Regressionsmodell mit der unabhängigen Variable Erhebungsjahr geschätzt (Modell 1), dann ergibt sich für männliche und weibliche Jugendliche sowie für die jungen Männer ein statistisch signifikant zurückgehender Trend des Rauschtrinkens. In der Gruppe der jungen Frauen gibt es keine statistisch signifikante Veränderung mit der Zeit.

**Table 4:** 

	Männliche Jugendliche		Weibliche Jugendliche		Junge Männer		Junge Frauen	
	Modell 1	Modell 2	Modell 1	Modell 2	Modell 1	Modell 2	Modell 1	Modell 2
Konstante	−1,279***	−0,986***	−1,777***	−1,768***	0,168***	0,207*	−0,966***	−0,831***
Bildung_(0|1)_	–	−0,282	–	0,162	–	0,025	–	0,037
Migrationshintergrund_(0|1)_	–	−0,888***	–	−0,404	–	−0,052	–	−0,454*
Bildung_(0|1)_ * Migrationshintergrund_(0|1)_	–	0,408	–	0,217	–	−0,157	–	−0,219
Jahr	−0,037***	−0,065***	−0,042***	−0,034	−0,038***	−0,036**	−0,011	−0,053**
Bildung_(0|1)_ * Jahr	–	0,039*	–	−0,013	–	0,005	–	0,049*
Migrationshintergrund_(0|1)_ * Jahr	–	0,049	–	−0,008	–	−0,079**	–	−0,017
Bildung_(0|1)_ * Migrationshintergrund_(0|1)_ * Jahr	–	−0,035	–	−0,043	–	0,054	–	0,019
BIC	8334,8	8328,1	5252,1	5274,2	19092,7	19007,8	13267,6	13113,0

*BIC* Bayes’sches Informationskriterium

^*^ *p* < 0,05, ^**^ *p* < 0,01, ^***^ *p* < 0,001

Modell 1: logit (Rauschtrinken = 1) = b_0_ + b_1_ (Erhebungsjahr-2007)

Modell 2: logit (Rauschtrinken = 1) = b_0_ + b_1_ (Bildung_(0|1)_) + b_2_ (Migrationshintergrund_(0|1)_) + b_3_ (Bildung_(0|1)_) (Migrationshintergrund_(0|1)_) + b_4_ (Erhebungsjahr-2007) + b_5_ (Bildung_(0|1)_) (Erhebungsjahr-2007) + b_6_ (Migrationshintergrund_(0|1)_) (Erhebungsjahr-2007) + b_7_ (Bildung_(0|1)_) (Migrationshintergrund_(0|1)_) (Erhebungsjahr-2007)

Durch die 0/1-Codierung von Bildung und Migrationshintergrund ergibt sich zum Beispiel für junge Frauen mit (Fach‑)Abitur und ohne Migrationshintergrund diese Regressionsgleichung: logit (Rauschtrinken = 1) = (−0,831 +0,037)  + (−0,053 +0,049) (Erhebungsjahr-2007) = −0,794–0,004 (Erhebungsjahr-2007)

Wird das Regressionsmodell um Bildung und Migrationshintergrund und deren Interaktionen mit dem Erhebungsjahr erweitert (Modell 2, Tab. 4), ergeben sich zusätzliche signifikante Effekte und differenziertere Trendverläufe.

**Figure 1:**
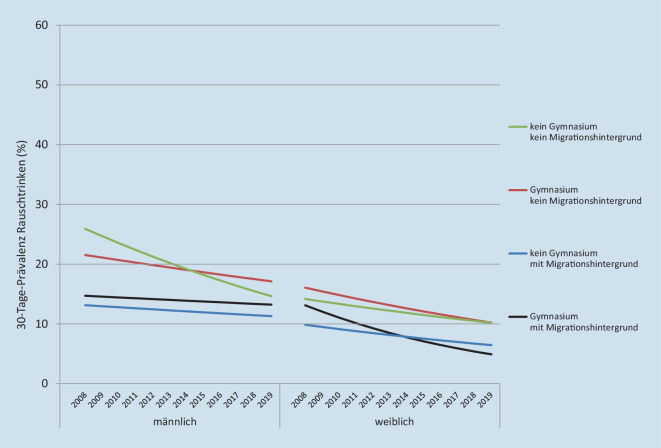

Im erweiterten Modell der männlichen Jugendlichen sind neben dem Erhebungsjahr der Migrationshintergrund und die Interaktion von Bildung und Erhebungsjahr statistisch signifikant. Der kleinere BIC-Wert deutet darauf hin, dass die Modellanpassung durch die Modellerweiterung etwas verbessert wird. Die auf Modell 2 beruhenden Trendverläufe zeigen, dass bei männlichen Jugendlichen ohne Migrationshintergrund Rauschtrinken weiter verbreitet ist als bei männlichen Jugendlichen mit Migrationshintergrund, in allen Gruppen das Rauschtrinken zurückgeht und dieser Rückgang in der Gruppe der männlichen Jugendlichen, die nicht das Gymnasium besuchen und keinen Migrationshintergrund haben, am stärksten ist (Abb. 1, linke Hälfte).

Die Modellerweiterung führt bei weiblichen Jugendlichen zu keiner Verbesserung. Außer der Konstanten ist kein Term der Regressionsgleichung statistisch signifikant. Auch der Einfluss des Erhebungsjahres ist knapp nicht signifikant (B = −0,034 mit *p* = 0,071). Das heißt, insgesamt verringert sich im Laufe der Zeit das Rauschtrinken weiblicher Jugendlicher (Modell 1) und zwar unabhängig von ihrer Bildung und ihrem Migrationshintergrund. Die Trendverläufe für die weiblichen Jugendlichen (Abb. 1, rechte Hälfte) liegen enger zusammen und unterscheiden sich in ihrem Rückgang weniger, als dies bei männlichen Jugendlichen der Fall ist.

**Figure 2:**
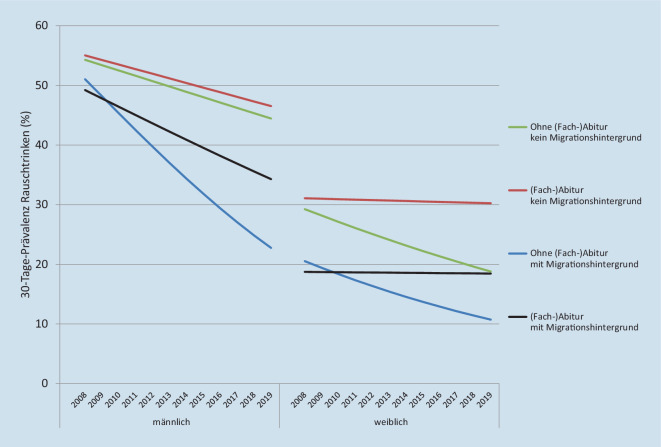

Im erweiterten Modell ist für die jungen Männer neben dem Erhebungsjahr die Interaktion von Migrationshintergrund und Erhebungsjahr statistisch signifikant. In den gruppenspezifischen Trends (Abb. 2, linke Hälfte) wirkt sich das so aus, dass das Rauschtrinken in allen Gruppen zurückgeht, sich aber bei Männern mit Migrationshintergrund stärker reduziert als bei jungen Männern ohne Migrationshintergrund.

Auch bei den jungen Frauen wird die zeitliche Entwicklung des Rauschtrinkens durch die Berücksichtigung von Bildung und Migrationshintergrund besser erklärt. In Modell 2 sind neben dem Erhebungsjahr der Migrationshintergrund und die Interaktion von Bildung und Erhebungsjahr statistisch signifikant. Die Interaktion zeigt auf, dass es in Abhängigkeit von der Bildung unterschiedliche Entwicklungen gibt (Abb. 2, rechte Hälfte). Während bei jungen Frauen ohne (Fach‑)Abitur das Rauschtrinken um etwa 10 Prozentpunkte deutlich zurückgeht, verändert es sich bei jungen Frauen mit (Fach‑)Abitur nicht. Das gilt auf unterschiedlichem Niveau für junge Frauen mit und ohne Migrationshintergrund.

## Diskussion

Dieser Beitrag stellt Trends des Rauschtrinkens für 12- bis 17-jährige Jugendliche und 18- bis 25-jährige junge Erwachsene in Deutschland dar. Neben Ergebnissen zu alters- und geschlechtsspezifischen Effekten wurde auch die Entwicklung in Abhängigkeit von Bildungsniveau und Migrationshintergrund untersucht. Die stärkere Differenzierung nach sozialen Merkmalen soll Aufschlüsse darüber geben, ob es in unterschiedlichen sozialen Teilgruppen unterschiedliche Entwicklungen im Rauschtrinken gibt. Dazu wurden Daten der Repräsentativerhebungen der BZgA aus dem Zeitraum 2008 bis 2019 ausgewertet.

Die Ergebnisse zeigen über alle Befragungen hinweg, dass sich mehr junge Erwachsene als Jugendliche, mehr männliche als weibliche junge Menschen und mehr junge Menschen ohne als mit Migrationshintergrund in einen Alkoholrausch trinken.

Insgesamt ist das Rauschtrinken im Jahr 2019 bei männlichen und weiblichen 12- bis 17-jährigen Jugendlichen geringer verbreitet als noch 2008. Andere aktuelle Studien in Deutschland stützen diesen Befund und zeigen, dass das Rauschtrinken unter Jugendlichen zurückgeht (z. B. [22, 25]). Auch international berichten Studien von sinkenden Zahlen des Alkoholkonsums bzw. des Rauschtrinkens bei Jugendlichen (z. B. in den USA [33], den Niederlanden [34] oder der Schweiz [35]).

Bei 18- bis 25-jährigen jungen Männern und Frauen in Deutschland entwickelt sich das Rauschtrinken in den vergangenen Jahren unterschiedlich. Während die 30-Tage-Prävalenz des Rauschtrinkens junger Männer im Zeitraum 2008 bis 2019 zurückgeht, zeichnet sich bei jungen Frauen insgesamt keine Veränderung ab. Auch der Epidemiologische Suchtsurvey (ESA) zeigt für den Zeitraum von 2006 bis 2018 keine wesentlichen Veränderungen im Rauschtrinken junger Frauen, aber bei jungen Männern. Der Epidemiologische Suchtsurvey zeigt aber auch, dass das Rauschtrinken unter jungen Erwachsenen im Zeitraum 2000 bis 2006 angestiegen ist und bei jungen Frauen derzeit deutlich über dem Niveau des Jahres 2000 liegt [36]. Im internationalen Kontext kommt eine Metaanalyse von Surveys aus den USA zu dem Schluss, dass in den letzten Jahren in der Gruppe der jungen Erwachsenen kein Rückgang des Rauschtrinkens zu beobachten ist [37].

Neben alters- und geschlechtsspezifischen Veränderungen des Rauschtrinkens zeigen sich Effekte in Abhängigkeit von Bildungsniveau und Migrationshintergrund. Diese stellen sich für männliche und weibliche Jugendliche und junge Erwachsene unterschiedlich dar. Bei männlichen Jugendlichen geht das Rauschtrinken insgesamt zurück und zwar besonders stark, wenn sie nicht das Gymnasium besuchen und keinen Migrationshintergrund haben. Bei weiblichen Jugendlichen geht das Rauschtrinken in allen Gruppen gleichermaßen zurück. Unter jungen Männern ist der Rückgang größer, wenn sie einen Migrationshintergrund haben. Bei den jungen Frauen gibt es eine Interaktion von Bildungsniveau und Zeit. Unabhängig vom Migrationshintergrund geht das Rauschtrinken bei jungen Frauen ohne (Fach‑)Abitur deutlich zurück. Bei jungen Frauen mit (Fach‑)Abitur verändert es sich nicht. Dieser Befund ist besonders hervorzuheben, weil bislang auf Basis von Analysen für die Gesamtgruppe davon ausgegangen wurde, dass sich das Rauschtrinken junger Frauen nicht verändert. Die hier vorgestellten Ergebnisse zeigen, dass bei differenzierter Analyse sozialer Merkmale es auch unter jungen Frauen eine Gruppe gibt, deren Entwicklung so günstig verläuft wie bei Jugendlichen und jungen Männern. Dies ist als ein deskriptiver Befund einzuordnen. Um sich einer ursächlichen Erklärung zu nähern, sind weitere Analysen erforderlich.

Auch im internationalen Kontext finden sich Studien, die in Abhängigkeit von sozialen Merkmalen unterschiedliche Entwicklungen des Alkoholkonsums aufzeigen. So kommt ein Literaturreview zum Alkoholkonsum junger Menschen zu dem Ergebnis, dass der Rückgang des Alkoholkonsums Jugendlicher weitestgehend in allen sozialen Schichten, Bildungs- und ethnischen Gruppen stattgefunden hat, aber nicht unbedingt in allen Gruppen gleich stark ausgefallen ist. Das Ausmaß der Veränderung variiert zudem je nach Land oder bezüglich der Indikatoren des Alkoholkonsums (z. B. Trinkhäufigkeit oder Häufigkeit des Rauschtrinkens; [27]).

Die hier dargestellten Befunde und Aussagen unterliegen insofern methodischen Einschränkungen, als dass sie auf Selbstauskünften der Befragten beruhen und zu einem gewissen Maß durch sozial erwünschtes Antwortverhalten verzerrt sein können, insbesondere wenn Befragte Rauschtrinken als sozial unerwünscht empfinden. Allerdings wurde nicht gefragt, ob man sich betrinkt, sondern ob man 5 Gläser Alkohol hintereinander getrunken hat, was eine neutralere Formulierung ist. Ein anderer Punkt betrifft die Fallzahlen, die sich durch die Binnendifferenzierung nach Bildungsniveau und Migrationshintergrund innerhalb der Alters- und Geschlechtergruppen ergeben. Dies führt in den einzelnen Untergruppen zu Punktschätzern mit teilweise großen Konfidenzintervallen und im Zeitverlauf zu stärkeren Schwankungen der geschätzten Prävalenzen. Die Regressionsanalysen modellieren die Veränderungen innerhalb der Alters- und Geschlechtergruppen über die gesamte Zeitspanne von 2008 bis 2019 nach Bildungsniveau und Migrationshintergrund gleichzeitig. Es wäre dennoch wünschenswert, sich mit Daten zukünftiger Erhebungen der Stabilität der hier gefundenen Trends zu versichern. Schließlich beruhen die Trendschätzungen wegen methodisch besserer Vergleichbarkeit der einzelnen Erhebungen ausschließlich auf den Festnetzstichproben. Vergleiche der Festnetzstichproben mit den Dual-Frame-Stichproben, die seit 2014 vorliegen, zeigen, dass mit den Festnetzstichproben bei jungen Erwachsenen, nicht aber bei Jugendlichen, die Schätzungen für die Verbreitung des Rauschtrinkens etwas geringer ausfallen als mit den Dual-Frame-Stichproben. Außerdem wurde für weibliche Befragte die Definition von Rauschtrinken im Sinne von 5 statt 4 oder mehr alkoholischen Getränken bei einer Gelegenheit verwendet, weil Daten nach der Definition von 5 oder mehr alkoholischen Getränken schon ab 2008 vorliegen. Die Verwendung der Definition von 4 oder mehr alkoholischen Getränken bei weiblichen Befragten führt zu höheren Prävalenzen. Die gemeinsame Darstellung beider Indikatoren [21] zeigt, dass diese auf etwas unterschiedlichem Niveau annähernd parallel verlaufen, sich mit beiden Definitionen sehr ähnliche Trendentwicklungen ergeben.

Trotz der beschriebenen Einschränkungen folgt aus den dargestellten Ergebnissen, dass Präventionsmaßnahmen altersgerecht und geschlechtersensibel gestaltet werden müssen und weitere soziale Unterschiede berücksichtigt werden sollten. Bei der Konzeption von lebenswelt- und settingbezogenen Präventionsmaßnahmen bietet es sich an, auch die unterschiedlichen Veränderungen im Rauschtrinken einzelner Gruppen, die anhand differenzierterer sozialer Merkmale identifiziert wurden, mit zu bedenken. Insbesondere junge Frauen mit höherem Bildungsniveau zeigen bislang keine Veränderung im Rauschtrinken. Daher ist es wichtig, insbesondere diese Gruppe mit Präventionsangeboten zu erreichen. Neben dieser gezielten Ansprache einzelner Gruppen bleiben aber weiterhin universelle Präventionsansätze von Bedeutung, die auf Wissen, Einstellungen und Verhalten aller und die Veränderung relevanter gesellschaftlicher Rahmenbedingungen und Verhältnisse zielen [38].

## Footnotes

1 In der „rauchfrei“-Jugendstudie 2007, in der der Migrationshintergrund zum ersten Mal erhoben wurde und die auch Fragen zum Alkoholkonsum beinhaltete, wurden 12- bis 19-Jährige befragt.

2 Im Alkoholsurvey 2012 wurde bei weiblichen Befragten der Frage zu 5 Gläsern oder mehr direkt eine Frage zu 4 Gläsern oder mehr vorangestellt. Dies führte zu unplausibel niedriger Angabe des Konsums von 5 Gläsern oder mehr, sodass die Angaben dieser Befragten nicht verwertet werden können. Ab 2014 wurde die Reihenfolge geändert, sodass für weibliche Befragte beide Maße vorliegen.
